# Management of musculoskeletal foot and ankle conditions prior to public-sector orthopaedic referral in South Australia

**DOI:** 10.1186/s13047-019-0331-4

**Published:** 2019-03-20

**Authors:** Tom P. Walsh, Linda R. Ferris, Nancy C. Cullen, Jared L. Bourke, Melissa J. Cooney, Chi K. Gooi, Christopher H. Brown, John B. Arnold

**Affiliations:** 1 School of Clinical Sciences, Faculty of Health, Queensland University of Technology, Kelvin Grove, Queensland 4059 Australia; 2 Department of Orthopaedics and Trauma, Queen Elizabeth Hospital, Central Adelaide Local Health Network, Woodville South, South Australia 5011 Australia; 3 Department of Orthopaedics, Noarlunga Hospital, Southern Adelaide Local Health Network, Noarlunga Centre, South Australia 5168 Australia; 4 Alliance for Research in Exercise, Nutrition and Activity, Sansom Institute for Health Research, School of Health Sciences, University of South Australia, Adelaide, South Australia 5000 Australia

**Keywords:** Podiatry, Orthopaedics, Body mass index, Quality of life

## Abstract

**Background:**

Foot and ankle pain is common in the Australian adult population. People with musculoskeletal foot and ankle conditions are often referred for surgical opinion, yet how patients are managed prior to referral is largely unknown. The aim of this study was to determine the characteristics and management of patients with musculoskeletal foot and ankle complaints prior to public-sector orthopaedic referral in South Australia.

**Methods:**

People with non-urgent foot or ankle complaints were recruited over a 12-month period from the waiting-lists of three tertiary hospitals in Adelaide, Australia. Participants completed a questionnaire on their medical history, duration and location of their foot or ankle complaint, diagnosis of their condition, previous treatment and medical imaging. The Manchester-Oxford Foot and Ankle Questionnaire, and the EuroQol-5D-5 L measured foot/ankle pain severity and health-related-quality-of-life (HRQoL). Descriptive statistics were generated for sample demographics, medical history and foot/ankle symptoms. Multivariable regressions were used to explore factors associated with foot/ankle pain severity and whether participants considered an operation necessary.

**Results:**

Two hundred and thirty-three adults returned questionnaires, with a survey response rate of 38.4% (66.1% female, median age 57.7 years IQR 18.5, BMI 29.3 kg/m^2^ IQR 8.7). Half of the participants had seen a podiatrist (52.8%), and 36.5% did not see any other health professional prior to orthopaedic referral. Sixty-five (27.9%) had not yet been given a diagnosis. BMI was positively associated with foot/ankle pain severity (β 0.48, 95% CI 0.05, 0.92), while HRQoL had a negative association (β − 0.31, 95% CI -0.45, − 0.18). Participants told by their GP that they may need an operation were significantly more likely to consider surgery necessary (OR 31.41, 95% CI 11.30, 87.35), while older people were less likely (OR 0.94, 95% CI 0.90, 0.98).

**Conclusions:**

More than one-third of the participants had not accessed allied-health care prior to specialist orthopaedic referral. Participants may consider their GPs opinion on the necessity of surgery compelling, and most expected to undergo surgery, but many couldn’t report their diagnosis. The discordance between the expectation of surgery and historically low surgical conversion rates suggests more work is necessary to improve the management of this group.

## Background

Musculoskeletal pain is a leading cause of burden of disease [1]. Foot and ankle pain affects 24 and 15% of adults aged 45 years and older, respectively [2]. Foot pain is associated with increased age, obesity, depression and reduced health-related quality of life (HRQoL) [3]. The back is the most frequently cited region for musculoskeletal pain [4], but the foot or ankle may account for nearly 10% of all musculoskeletal consultations with general practitioners (GPs) [5]. The management of foot or ankle pain typically involves non-surgical interventions in the first instance, which can be provided by GPs, podiatrists or other health professionals. Indeed, there is good evidence to support the use oral analgesics, exercises, orthoses and corticosteroid injections for various musculoskeletal foot or ankle conditions [6–10], but an actual or apparent failure in non-surgical management may prompt referral to orthopaedic foot/ankle surgeons for opinion and intervention. Gaining insight into the non-surgical management prior to surgical referral is important, as when a referral is made to orthopaedic surgeons in Australian public hospitals, it triggers a lengthy process that commonly does not result in an operation [11, 12].

Patients referred to a public hospital are usually characterised as urgent, semi-urgent or non-urgent by the orthopaedic surgeon, which dictates when they will be seen. This stratification is not necessarily based on a patient’s level of pain but rather on the diagnosis and history given by the referring GP. Patients considered non-urgent do not have life- or limb-threatening complaints, but they commonly have persistent, disabling foot/ankle pain. Despite often severe pain, and because of a high-demand and the relatively low number of foot/ankle surgeons in the public-sector in Australia, it is not unusual for a patient with a foot/ankle complaint to wait over 12-months for an initial appointment, with one study reporting patients may wait 2–3 years before being seen [11]. Whilst lengthy waiting times are a defining feature of orthopaedic management for a variety of musculoskeletal complaints in the public-sector, a low surgical conversion rate for foot/ankle complaints questions the value of the current process. The lengthy waiting period also highlights the importance of ensuring patients are given a diagnosis prior to referral, to allow them to optimise the management of their condition (either by themselves, or with another health provider) while they wait to be seen.

Some health networks have implemented innovative management strategies in response to the expansion of waiting-lists and the poor surgical conversion rate. Podiatrists, providing non-surgical interventions or education, working with an orthopaedic department have efficiently discharged patients from orthopaedic waiting-lists across Australia [11, 13, 14]. The success of these clinics is important, but it raises further questions as to why these patients were referred to an orthopaedic department in the first place. If patients are provided with practical, evidence-based treatments prior to referral, the discordance between referral and surgical conversion should be low. A recent analysis of the management of foot and ankle osteoarthritis by GPs in Australia identified a reliance on pharmacological management and relatively low use of lifestyle advice and allied health referral [15]. Furthermore, a recent study of patients with back pain found that a significant number of patients do not undergo evidence-based treatments prior to referral to an orthopaedic unit [16]. As in patients with back pain, referral to orthopaedic surgeons for foot/ankle complaints without non-surgical management, may be responsible for inflating the number of patients on a surgical specialty’s waiting list. To the authors’ knowledge, there are no prior studies investigating the characteristics and prior management of musculoskeletal foot/ankle complaints in adults awaiting specialist orthopaedic opinion in the public-sector in Australia.

The referral to an orthopaedic department may be due to the lack of access to podiatrists, or due to the persistence of the condition, which both the GP and the patient think would benefit from operative intervention. Given the complexities and heterogeneity of persistent pain, using the severity of symptoms as an indicator for surgical intervention may be misguided, but somewhat understandable as both parties seek a solution. Orthopaedic surgeons spend limited time in Australian public outpatient clinics [17], and given the prolonged waiting times, along with poor surgical conversion rates, understanding how patients are managed prior to referral is important, particularly if improvements can be made in general practice or by allied health professionals (AHPs). The aim of this cross-sectional study was therefore to determine how patients with musculoskeletal foot/ankle complaints are managed prior to referral, quantify both their foot/ankle pain and HRQoL, and to improve our understanding of patient expectations.

## Methods

### Participant recruitment

Participants were recruited consecutively over a 12-month period, from 1st November 2017 to 31st October 2018. All participants were recruited from the waiting-lists of the three major tertiary hospitals in Adelaide, Australia, which provide elective foot/ankle orthopaedic surgery. These hospitals provide services to people from the Northern, Central and Southern Adelaide Local Health Networks, along with Country Health SA Local Health Network. Potential participants were identified shortly after referral from their GP (within 3-months) and were posted a letter of invitation, a participant information sheet and a copy of the survey. Basic demographic characteristics (age, gender, socio-economic status) of non-responders was also recorded to determine the presence and potential impact of self-selection bias on the survey results. This study was given ethical approval by the Central Adelaide Local Health Network Human Research Ethics Committee (Study ID Q20180109).

### Inclusion and exclusion criteria

All patients on the orthopaedic waiting-list with foot/ankle complaints considered as non-urgent (category 3) were invited to participate in this study. Patients were excluded from invitation if they were categorised as either urgent (category 1) or semi-urgent (category 2) by the foot/ankle orthopaedic consultants. Only non-urgent cases were considered by this study given the known low surgical conversion rate and long waiting periods experienced by this group.

### Anthropometry and medical history

Participants were asked to self-report their height and body weight. Information about medical conditions was gathered and whether the participants were current or ex-smokers was also collected.

### Expectations and understanding of their foot/ankle complaint

Participants were asked to report if; i) they had been given a diagnosis, ii) they knew the name of the diagnosis, iii) their GP had told them that they may need an operation and, iv) they thought they needed an operation.

### Previous medical imaging, referral and non-surgical intervention

Participants were asked to report previous referrals and interventions including; i) referral for medical imaging (plain film radiograph, diagnostic ultrasound, computed tomography, magnetic resonance imaging, nuclear medicine scan), ii) referral to another health professional about their foot/ankle complaint (podiatrist, physiotherapist, chiropractor, pharmacist, other) and, iii) non-surgical interventions they received (directed by their GP or other health care professional).

### Foot/ankle pain

The duration of symptoms was recorded and the region of foot/ankle pain was documented using a foot/ankle manikin [18]. Foot/ankle pain and disability was assessed using the Manchester-Oxford Foot and Ankle Questionnaire (MOXFQ), which is a patient-reported outcome measure that assesses the impact of region-specific dimensions on quality of life. The MOXFQ is a reliable and valid 16-item questionnaire that comprises three separate underlying dimensions: walking/standing problems (seven items), foot pain (five items), and social interaction (four items) [19]. Item responses are each scored from 0 to 4, with 4 representing the most severe state. Raw scores are converted to a transformed score on a 0–100 scale, with 100 being the most severe pain. The MOXFQ summary score (MOXFQ-index) [20] was used in the regression analyses assessing foot/ankle pain severity.

### Health-related quality of life

Participants were asked to complete the EuroQoL-5-Dimensions-5-Levels (EQ-5D-5 L) to measure overall HRQoL. The EQ-5D-5 L is a descriptive system comprising of the following five dimensions: mobility, self-care, usual activities, pain/discomfort and anxiety/depression. Each dimension has five levels: no problems, slight problems, moderate problems, severe problems and extreme problems. The participants were asked to indicate their health state by ticking the box next to the most appropriate statement in each of the five dimensions. The EQ visual analogue scale (EQ VAS) records the participant’s self-rated health on a vertical visual analogue scale where the endpoints are labelled ‘best health you can imagine’ and ‘worst health you can imagine’. The EQ VAS can be used as a quantitative measure of health outcome that reflects the participant’s own judgement and is graded from 0 to 100, with score of 0 being the worst imaginable health state. Both the EQ-5D-5 L and EQ VAS have undergone extensive, structured psychometric development and been previously used to assess HRQoL in people undergoing foot/ankle surgery [21, 22].

### Socio-economic status

Socio-economic disadvantage (SED) is a known factor for higher levels of musculoskeletal pain [23]. The SED was estimated from the participant’s postcode, using the Index of Relative Socio-economic Disadvantage (IRSD) from the Australia Bureau of Statistics [24], which is graded from 1 to 10, with a score of 1 being the most disadvantaged.

### Data analysis

Descriptive statistics were used to summarise the demographic and clinical characteristics of the participants, their diagnosis and beliefs regarding need for surgical intervention, prior treatments and investigations, their foot/ankle pain and HRQoL. All data distributions were checked for normality via the inspection of histograms and the Shapiro-Wilks test prior to inferential statistical analysis. As age and SED were not normally distributed in those that did and did not respond to the survey, the Mann-Whitney *U* test was used to assess for between-group differences and the chi-squared test was used to assess for between-group differences in gender. Multivariable linear regression was used to determine if foot/ankle pain severity (outcome) was associated with age, gender, BMI, HRQoL, SED, depression or duration of foot/ankle pain. Standard homoscedasticity and normality checks of residuals were carried out. Multivariable binary logistic regression was used to determine if age, gender, BMI, HRQoL, depression, severity of foot/ankle pain, duration of foot/ankle pain, or if the GP told the participant they may need an operation were associated with the participant considering an operation necessary. The final model was tested for goodness of fit using the Hosmer and Lemeshow goodness-of-fit test. If the value of the chi-squared statistic in this test is low, the *p*-value is not significant and indicates that the model is a good fit for the data [25]. Results for continuous outcomes are summarised with regression coefficients (β) and dichotomous outcomes are presented as adjusted odds ratios (OR) with 95% confidence intervals (95% CI). In all analyses, a p-value (two-sided) less than 0.05 was deemed to be statistically significant. All analyses were conducted using SPSS v25 (IBM SPSS Statistics, Armonk, NY, USA).

### Sample size calculation

The study was exploratory in nature but estimates were made a priori about the number of variables to be included in the multivariable models. A conservative estimate of 20 participants per independent variable was accepted in the linear [26] and logistic regression models [27] which given six and seven planned variables in each of the models, required at least 140 participants for this study.

## Results

### Study participants

A total of 606 questionnaires were posted to patients, with 233 participants completing the questionnaires, yielding a response rate of 38.4%. The Central Adelaide Local Health Network had the highest participation rate with 51/90 (56.7%) of patients responding to the survey. This was followed by the Northern and Southern Adelaide Local Health Networks, with 72/201 (35.8%) and 110/315 (34.9%), respectively. There were no significant between-group differences for the number of women 154 (66.1%) versus 245 (65.7%), *X*^*2*^ = 0.011, *p* = 0.917 or SED with a median (interquartile range (IQR)) of 4 (4) versus 4 (4), *p* = 0.537 comparing those who participated in the study and those who did not. The participants, however, were significantly older than the people that did not participate with a median (IQR) age of 57.7 (18.5) years versus 53.1 (22.2) years, *p* <  0.001, respectively. The participants had a median (IQR) BMI of 29.3 (8.7) kg/m^2^ and reported symptoms for a median (IQR) of 55.0 (96.0) months. A total of 61 (26.2%) participants had previously had foot/ankle surgery. Over 20% of the participants had a score of 1 out of 10 for SED, and 75% had a score of 5 or less. Only 4 participants were rated as scoring 10 out of 10 for SED.

### Co-morbidities and health-related quality of life

The most prevalent co-morbidity was back pain (56.7%), followed by osteoarthritis (49.4%), hypertension (39.1%) and depression (33.9%). The frequency of all of the co-morbidities recorded are reported in Table 1. Thirty-six participants were current cigarette smokers, 81 previously smoked and 116 had never smoked. The median (IQR) of the EQ VAS was 65.0 (30.0) points, with details of the five dimensions assessed with the EQ-5D-5 L reported in Table 2.

**Table 1:** Participant characteristics (*n* = 233)

Characteristic	median (IQR)
Age, years	57.7 (18.5)
Gender, no. women (%)	154 (66.1)
Height, cm	168.0 (15.0)
Weight, kg	84.0 (29.5)
BMI, kg/m^2^	29.3 (8.7)
SED	4 (4)
Duration of symptoms, months	55.0 (96)
History of foot surgery, n (%)	61 (26.2)
EQ VAS	65.0 (30.0)
MOXFQ domains	
Walking/standing	75.0 (42.9)
Pain	70.0 (30.0)
Social interaction	56.3 (43.7)
MOXFQ-index	68.8 (31.3)
Co-morbidity	n (%)
Heart disease	20 (8.6)
Hypertension	91 (39.1)
Lung disease	16 (6.9)
Diabetes	28 (12.0)
Anaemia or other blood disease	6 (2.6)
Kidney disease	5 (2.1)
Liver disease	4 (1.7)
Depression	79 (33.9)
Cancer	9 (3.9)
History of stroke	8 (3.4)
Osteoarthritis	115 (49.4)
Back pain	132 (56.7)
Rheumatoid arthritis	44 (18.9)
Other	52 (22.3)
participant knowledge and expectations	n (%)
Diagnosis given to participant	168 (72.1)
Diagnosis known by participant	145 (62.2)
Told by GP that they may need operation ^a^	161 (71.2)
participant thinks that they need an operation ^b^	165 (77.1)

^a^ 7 participants did not answer, ^b^ 19 participants did not answer

Abbreviations: *BMI* body mass index, *cm* centimetres, *EQ VAS* EuroQoL visual analogue scale, *GP* General Practitioner, *IQR* interquartile range, *kg* kilograms, *m* metres, *MOXFQ* Manchester-Oxford Foot and Ankle Questionnaire, *SED* socio-economic disadvantage

**Table 2:** Health-related quality of life measured by EuroQoL-5D-5 L (*n* = 232)

Dimension	Level				
	no problems	slight problems	moderate problems	severe problems	extreme problems
Mobility	25 (10.7)	55 (23.6)	81 (34.8)	69 (29.6)	2 (0.9)
Self-care	162 (69.5)	36 (15.5)	24 (10.3)	8 (3.4)	2 (0.9)
Usual activity	38 (16.3)	63 (27.0)	78 (33.5)	44 (18.9)	9 (3.9)
Pain / discomfort	3 (1.3)	33 (14.2)	95 (40.8)	78 (33.5)	23 (9.9)
Anxiety / depression	74 (31.8)	69 (29.6)	48 (20.6)	25 (10.7)	15 (6.4)

Values are n (%)

Abbreviations: *5D-5 L* 5-Dimensions-5-Levels

### Foot/ankle pain location and severity

The median (IQR) foot pain severity as measured by the MOXFQ-index was 68.8 (31.3) points. The three domains, other than MOXFQ-index, measured by the MOXFQ are reported in Table 1. The location of foot and ankle pain was reported across all regions of the foot and ankle manikin, with the three most common regions being the lesser toes (42.1%), the midfoot (38.8%) and the great toe (37.3%), the distribution of pain in the left and right foot are reported in Table 3. Bilateral foot pain was present in 94 participants. Multivariable linear regression found that BMI was positively associated with foot pain severity (β = 0.48, 95% CI 0.05 to 0.92, *p* = 0.030) and HRQoL was negatively associated with foot pain severity (β = − 0.31, 95% CI -0.45 to − 0.18, *p* <  0.001) (Table 4). The *R*^*2*^ value for the model was 0.18, indicating that 18% of the variance in the MOXFQ-index pain score could be explained by the independent variables.

**Table 3:** Region of foot/ankle pain experienced by the participants (*n* = 233)

Region	Left	Right
First metatarsophalangeal joint	71 (30.5)	77 (33.0)
Hallux	49 (21.0)	57 (24.5)
Great toe	81 (34.8)	93 (39.9)
Lesser toes	95 (40.8)	101 (43.3)
Plantar forefoot	67 (28.8)	61 (26.2)
Midfoot	99 (42.5)	82 (35.2)
Medial arch	40 (17.2)	37 (15.9)
Ankle	65 (27.9)	63 (27.0)
Plantar heel	44 (18.9)	43 (18.5)
Posterior heel	54 (23.2)	49 (21.0)

Values are n (%)

**Table 4:** Multivariable linear regression investigating factors associated with foot/ankle pain severity (MOXFQ-index)

	β-coefficients (95% CI)	*P* value
Age	−0.12 (−0.31 to 0.08)	0.249
Body mass index	0.48 (0.05 to 0.92)	0.030
Depression	−2.09 (−8.19 to 4.02)	0.501
Duration of symptoms	0.02 (−0.01 to 0.04)	0.114
Gender (female)	−1.38 (−7.00 to 4.23)	0.629
Heath-related quality of life (EQ VAS)	−0.31 (− 0.45 to − 0.18)	< 0.001
Socio-economic disadvantage	− 0.87 (− 1.90 to 0.16)	0.099

Abbreviations: *CI* confidence interval, *EQ VAS* EuroQoL visual analogue scale, *MOXFQ* Manchester-Oxford Foot and Ankle Questionnaire

### Participant expectations and knowledge of diagnosis

Sixty-five participants (27.9%) had not been given a diagnosis and a further 23 participants who had been given a diagnosis could not report the name. One-hundred-and-sixty-one (71.2%) participants reported that their GP thought they may need an operation and 165 (77.1%) participants thought they needed an operation. Multivariable binary logistic regression found that the strongest correlate for a participant considering that an operation was necessary, was if their GP told them that they may need an operation (OR 31.41, 95% CI 11.30 to 87.35, *p* <  0.001). Age was also a significant correlate, although this was negatively associated with the participant thinking surgery was necessary (OR 0.94 (95% CI 0.90 to 0.98, *p* = 0.001). Hosmer and Lemeshow goodness-of-fit *X*^2^ = 4.344, df = 8, *p* = 0.825 (Table 5).

**Table 5:** Multivariable binary logistic regression investigating factors associated with the expectation of an operation by the participants

	Odds ratio (95% CI)	*P* value
Age	0.94 (0.90 to 0.98)	0.001
Body mass index	0.98 (0.91 to 1.06)	0.641
Depression	0.36 (0.11 to 1.14)	0.082
Duration of symptoms	1.00 (1.00 to 1.01)	0.190
Gender (female)	0.63 (0.22 to 1.77)	0.379
Heath-related quality of life (EQ VAS)	1.00 (0.98 to 1.03)	0.728
Foot/ankle pain severity (MOXFQ-index)	1.02 (0.99 to 1.05)	0.142
Told by GP that they may need an operation	31.41 (11.30 to 87.35)	< 0.001

Abbreviations: *CI* confidence interval, *EQ VAS* EuroQoL visual analogue scale, *GP* General Practitioner, *MOXFQ* Manchester-Oxford Foot and Ankle Questionnaire

### Previous referral to health professionals, medical imaging and treatment

Other than seeing their GP, half of the study participants (52.8%) had seen a podiatrist and 23.2% had seen a physiotherapist prior to referral. These professions accounted for the majority of the other health professionals consulted prior to referral. Eighty-five (36.5%) participants did not see any other health professional prior to orthopaedic referral.

General practitioners instigated a variety of pharmacological and non-pharmacological treatments prior to referral. The most common treatment was paracetamol which was used in 123 participants (52.8%), followed by a change of footwear (46.8%), oral non-steroidal anti-inflammatory drugs (NSAIDs) (45.5%), exercises (38.6%), foot orthoses (35.6%) and topical NSAIDs (35.2%). Oral codeine/opioids was not used as frequently as a number of other treatments but was still reported to be provided to 26.6% of the participants.

The treatments instigated by health professionals other than the participant’s GP favoured non-pharmacological measures. Exercises, orthoses and a change in footwear were prescribed for 66 (44.6%), 66 (44.6%) and 63 (42.6%) participants, respectively. A detailed assessment of the treatments instigated prior to referral are reported in Table 6.

**Table 6:** Previous treatments provided to the participants prior to referral to public-sector orthopaedic department

	General practitioner initiated, (n = 233)	Other health professional initiated, (*n* = 148)
Analgesia		
Paracetamol	123 (52.8)	44 (29.7)
Oral NSAIDs	106 (45.5)	37 (25.0)
Codeine/opioids	62 (26.6)	22 (14.9)
Topical NSAIDs	82 (35.2)	32 (21.6)
Corticosteroid injection	63 (27.0)	30 (20.3)
Change of footwear	109 (46.8)	63 (42.6)
Foot orthoses	83 (35.6)	66 (44.6)
Exercises	90 (38.6)	66 (44.6)
Ankle brace	33 (14.2)	16 (10.8)
Walking aid (stick or frame)	31 (13.3)	13 (8.8)

Values are n (%)

Abbreviations: *NSAIDs* non-steroidal anti-inflammatory drugs

Most participants had undergone a plain film radiograph examination prior to referral (78.1%), over half had had an ultrasound (53.6%) and 14.2% had had magnetic resonance imaging. Nuclear bone scanning and computed tomography scanning were the less commonly used imaging modalities prior to referral (Table 7). Thirty-five (15.0%) participants were not referred for any medical imaging prior to orthopaedic referral.

**Table 7:** Referrals made by the participants general practitioner prior to referral to public-sector orthopaedic department (*n* = 233)

	n (%)
Medical imaging	
Plain film radiography	182 (78.1)
Ultrasound	125 (53.6)
Computed tomography	28 (12.0)
Nuclear medicine	23 (10.0)
Magnetic resonance imaging	33 (14.2)
Other health professional	
Podiatrist	123 (52.8)
Physiotherapist	54 (23.2)
Chiropractor	13 (5.6)
Pharmacist	18 (7.7)
Other	5 (2.1)

## Discussion

This study is the first to comprehensively evaluate the characteristics and prior management of patients on waiting lists with non-urgent orthopaedic foot/ankle complaints in Australia. It found a high prevalence of co-morbidity and the majority of the participants were socio-economically disadvantaged. Non-surgical treatment and referral to other health professionals was given to half of the participants, with oral analgesics and podiatrists being the most commonly used, respectively. Foot/ankle pain severity was positively associated with BMI and negatively associated with HRQoL, and foot/ankle pain had been present for more than two-years in over three-quarters of the participants. The majority of participants thought they needed an operation, and the strongest correlate being if their GP had told them that this may be necessary.

These data suggest that patients are being provided with a variety of therapies prior to referral, with both pharmacological and non-pharmacological therapies instigated by GPs and other health professionals. Over 50% of the participants are accessing podiatrists prior to referral, but over 35% of the participants did not receive a referral to any other health professional prior to their orthopaedic referral. This may explain why a number of studies report a high discharge rate (> 50%) when patients with non-urgent foot/ankle complaints are initially triaged by podiatrists in public hospitals [11–13]. These results suggest that the introduction of podiatrists to ensure appropriate non-surgical therapies are implemented prior to evaluation by a surgeon may be of benefit. Moreover, given the association of BMI and HRQoL [28], which were both associated with foot/ankle pain severity in this study, further evaluation of the early introduction of non-traditional members of the foot/ankle orthopaedic team, such as dieticians and psychologists, could be used to address factors beyond the scope of orthopaedic surgeons, podiatrists and physiotherapists.

There was a clear, positive association between foot pain severity and BMI, after adjusting for multiple confounding variables. Over 70% of the participants were classified as overweight and over 40% were classified as obese. Given people often over-estimate their height and under-estimate their weight in self-report [29], these figures likely under-estimate their prevalence. The association of obesity and foot pain has been well described and acknowledged [30] and whilst increased mechanical loading is the most obvious link to foot pain, the mechanisms underpinning this association may indeed be related to the non-mechanical influences of adiposity [31, 32]. There is an association between increased pain sensitivity and obesity [33], and obesity has also recently been found to have a causal role in developing depression [34], another known predictor of pain [35]. Given the cross-sectional nature of the study, it is not possible to determine cause and effect role of BMI and foot/ankle pain severity, nevertheless, it is well understood that there is a bidirectional relationship between chronic pain and obesity [36] and therefore addressing both factors may be necessary to effectively reduce symptoms.

It is unclear if the strong association between the patient’s and GP’s belief about the need for surgery is driven by both parties seeking a solution for a persistent problem, or if the risks and benefits of surgery have been understood and considered, and conclusions drawn that this is the most suitable option. In Australia, GPs may feel that surgeons are more capable in managing musculoskeletal foot/ankle pain, and a study found that GPs are significantly more likely to refer patients with foot/ankle osteoarthritis to an orthopaedic surgeon than to an AHP [15], but it is unknown if this is similar for other musculoskeletal foot/ankle conditions. The design of the Australian healthcare system, which is predicated on unfettered access to GPs and publicly-funded specialist consultation and intervention, may be responsible for encouraging referral from GPs to surgeons. Whereas the access to publicly-funded AHPs is more convoluted and constrained, and there can be substantial heterogeneity in the number and types of consultations and interventions available to patients.

Irrespective, given the historically low rates of surgical conversion for non-urgent patients in Australian public hospitals [11–13], there is a disconnect between what the patient believes will happen and what eventuates. Indeed, patients with foot/ankle pain referred to Australian public hospital outpatient clinics may be most appropriately prepared by being informed that they are *unlikely* to receive surgery which may assist in managing expectations. Less constrained, and early access to AHPs may also assist in giving practical, evidence-based advice and management to this patient group.

The management of expectations is a key factor in managing musculoskeletal pain, particularly when pain may be a manifestation of other conditions or complaints. The participants in this study had a high prevalence of features known to predict or amplify chronic pain, notably depression and obesity and therefore resolving pain without addressing these factors may be challenging. Patients referred for evaluation of musculoskeletal complaints can often struggle to accept or acknowledge that there may be strong non-nociceptive drivers related to their foot/ankle pain. Furthermore, previous studies have found people with chronic pain may have compromised executive function [37] and therefore may have difficulty in analysing risk, reward and benefit, which further complicates their management. This may contribute to the over-representation in the number of participants considering that an operation is necessary, without a thorough understanding of the expected benefit and inherent risks a surgical procedure entails. The focus of therapies at a local (foot/ankle) level, surgery in particular, may be misguided and ultimately unsatisfying, if patients see surgery as a panacea for any and all foot/ankle pain.

This study does have limitations that must be considered. Firstly, the response rate is respectable for this type of study, but given it was less than 40% it may not be representative of all patients on the waiting-list. Secondly, given data were only collected from one state in Australia, these data may not be reflective of other regions and, furthermore, given the study is completed in the public-sector the results may not be generalisable to people seen in the private sector. This study did also not report or measure more detailed foot and ankle characteristics typically performed in clinical assessments, as this would involve a significantly larger investment of time and resources. Furthermore, self-report on management data may be affected by recall bias and specific details on each case would have been desirable, but outside of the scope of this study. Finally, given the cross-sectional nature of this study, how the foot/ankle pain and patient expectations change over time cannot be reported.

This study does have a number of strengths. It provides a comprehensive assessment of foot/ankle pain, HRQoL, previous treatments and imaging, along with patient expectations. This is important for this group of patients as they can be on waiting lists for an extended period. Future work in this area may focus on evaluating the impact of strategies designed to provide patients with early access to appropriately skilled health professionals to offer a timely diagnosis, provide appropriate expectations, and finally to address factors that are associated with foot/ankle pain severity, such as BMI and HRQoL.

## Conclusion

In conclusion, this study has found that patients referred to orthopaedic departments in public hospitals with non-urgent foot/ankle complaints do undergo some non-surgical therapy prior to referral and most have medical imaging, but there is substantial heterogeneity in what is provided. Patients may not be well prepared or well advised about both the diagnosis and prognosis of their foot/ankle complaint, as most participants expect to have an operation, yet more than 25% did not know their diagnosis. Body mass index and HRQoL are both related to foot/ankle pain severity and further study to address and improve these findings may be prudent, particularly as waiting-lists continue to grow.

## Abbreviations

5D-5 L: 5-Dimensions-5-Levels
AHP: Allied Health Professional
BMI: Body mass index
CI: Confidence interval
cm: Centimetre
EQ: EuroQoL
GP: General Practitioner
HRQoL: Health-related quality of life
IBM: International Business Machines
IQR: Interquartile range
IRSD: Index of Relative Socio-economic Disadvantage
MOXFQ: Manchester-Oxford Foot and Ankle Questionnaire
NSAIDs: Non-steroidal anti-inflammatory drugs
NY: New York
SED: Socio-economic disadvantage
SPSS: Statistical Package for the Social Sciences
USA: United States of America
VAS: Visual analogue scale
